# Predicting and understanding human action decisions during skillful joint-action using supervised machine learning and explainable-AI

**DOI:** 10.1038/s41598-023-31807-1

**Published:** 2023-03-27

**Authors:** Fabrizia Auletta, Rachel W. Kallen, Mario di Bernardo, Michael J. Richardson

**Affiliations:** 1 School of Psychological Sciences, Faculty of Medicine, Health and Human Sciences, Macquarie University, Sydney, NSW Australia; 2 Department of Engineering Mathematics, University of Bristol, Bristol, UK; 3 Center for Elite Performance, Expertise and Training, Macquarie University, Sydney, NSW Australia; 4 Department of Electrical Engineering and Information Technology, University of Naples, Federico II, Naples, Italy; 5 Scuola Superiore Meridionale, Naples, Italy

**Keywords:** Human behaviour, Computational science

## Abstract

This study investigated the utility of supervised machine learning (SML) and explainable artificial intelligence (AI) techniques for modeling and understanding human decision-making during multiagent task performance. Long short-term memory (LSTM) networks were trained to predict the target selection decisions of expert and novice players completing a multiagent herding task. The results revealed that the trained LSTM models could not only accurately predict the target selection decisions of expert and novice players but that these predictions could be made at timescales that preceded a player’s conscious intent. Importantly, the models were also expertise specific, in that models trained to predict the target selection decisions of experts could not accurately predict the target selection decisions of novices (and vice versa). To understand what differentiated expert and novice target selection decisions, we employed the explainable-AI technique, SHapley Additive explanation (SHAP), to identify what informational features (variables) most influenced modelpredictions. The SHAP analysis revealed that experts were more reliant on information about target direction of heading and the location of coherders (i.e., other players) compared to novices. The implications and assumptions underlying the use of SML and explainable-AI techniques for investigating and understanding human decision-making are discussed.

## Introduction

Performing tasks with other individuals is an essential part of everyday human life. Such behavior requires that co-actors reciprocally coordinate their actions with respect to each other and changing task demands^1–4^. Pivotal to the structural organization of such action is the ability of co-actors to effectively decide how and when to act^5^, with robust decision-making often differentiating expert from non-expert performance^6^. This is true whether one considers the simple activity of family members loading a dishwasher together^7–9^, or the more complex activities performed by elite athletes during team sports^10,11^ or soldiers during high-stakes military operations^12^.

In contrast to practical reasoning or deliberative decision-making, where an actor extensively evaluates all possibilities to determine the optimal action, the decision-making that occurs during skillful action is typically fast-paced and highly context dependent^13–16^, with actors spontaneously adapting their actions to achieve task goals as “best as possible”^17^. Indeed, the effectiveness of action decisions during skillful behavior is a function of an actor’s level of situational awareness^18,19^, with task expertise reflecting the attunement of an actor to the information that specifies what action possibilities (optimal or sub-optimal) ensure task completion^20–22^. Developing a comprehensive understanding of decision-making during skillful behavior therefore rests on the ability of researchers and practitioners to identify what task information underlies effective decision-making performance. Key to achieving this, is developing decision-making models that can not only predict the action decisions of human actors during skillful action, but also help to identify what differentiates expert from non-expert performance. Motivated by these challenges, the current study proposes the use of state-of-the art Supervised Machine Learning (SML) and explainable AI (Artificial Intelligence) techniques to model, predict and explicate the action decisions of expert and novice pairs performing a fast-paced joint-action task. Specifically, for the first time in the literature, we show how using these computational techniques renders it possible to uncover the crucial information that co-actors exploit when making action decisions in joint-action (and individual) task contexts.

### Supervised machine learning

The application of Machine Learning (ML) techniques has rapidly increased over the last decade. For example, ML is now integral to modern image and speech recognition^23–26^, scientific analysis^27,28^, digital manufacturing and farms^29,30^, financial modeling^31^, and online product, movie and social interest recommendations^32–34^. In many contexts, ML models are trained via SML, whereby computational models learn to correctly classify input data, or predict future outcomes states from input data, by leveraging coded, realword training samples^35,36^. Training samples include representative task data (e.g. images, sounds, motion data) that have been labeled with the correct data class or outcome state. These training samples are then used to build an approximate model of how the input data (e.g., pixels from an image) map to the correct output label (e.g., cat or dog)^37,38^. Following training, the efficacy of the model is then tested against data not supplied during training, with effective models able to generalize the learned input–output associations to unseen data.

### Artificial neural and long short-term memory networks

SML models can be realized in numerous ways, for instance, using decision trees^39,40^, support vector machines^41,42^ or, of particular importance here, Artificial Neural Networks (ANNs). In general, ANNs are a composition of elementary units, *nodes*, that are grouped in interconnected *layers*, where nodes have different activation functions and the connections between nodes can have different weights. A typical ANN includes an input and an output layer, with 1 or more “hidden layers” in between (with deeper ANNs having more hidden layers). Training an ANN to model input–output associations via SML requires finding the combination of network parameters (weights and biases) that map input data to the correct output class or state prediction. This is achieved by iteratively evaluating the error between the correct and predicted output of the ANN (via a *loss function*) and adjusting the network parameters to minimize this error using a process called *back-propagation* (see e.g.^43^ for more details).

There are various types of ANNs. Of relevance here, is an ANN known as a *Long Short-Term Memory* (LSTM) network, which is a form of recurrent neural network that in addition to feed-forward connections among node layers also includes feedback connections. These feedback connections enable the ANN to process and retain information about sequences of consecutive inputs^44,45^. Accordingly, LSTMs are commonly used in time-series prediction tasks^46–51^, where the processing of both past and present input states is required to correctly predict future states. LSTMs are applicable to predicting human behavior^52–54^, as human action decisions are based on the assessment of dynamical (time varying) task information^10,22^ and, thus, the prediction of future state behavior or action decisions requires processing sequences of task relevant state input data.

### Explainable AI

Despite the increasing utility and effectiveness of ANNs in recent years^24,25,33,55^, the large number of connection weights and the non-linearity of the activation functions within ANNs, their generalization limits and complex decision boundaries, particularly in Deep-ANNs, makes it difficult to directly access how input features relate to output predictions. For this reason, ANNs are often referred to as “black-box” models. However, a desire to better understand and interpret the validity of ANNs and other black-box models, as well as the growing demand for more ethical and transparent AI systems^56^, has resulted in a renewed interest in the application and development of explainable-AI techniques^50,57–60^ such as LIME^61^, DeepLIFT^62^, LRP^63,64^ and, more recently, SHapley Additive exPlanation (SHAP)^65,66^, which we employ here.

These techniques make the internal processes of a black-box model understandable by deriving linear explanation functions of the effects that input features have on output states. For example, the SHAP algorithm pairs each input feature with a SHAP value. The higher the SHAP value, the greater the influence that feature has on an output state. Given that SHAP values are locally accurate, one can derive a measure of global feature importance by calculating the average importance of a feature over the test set used to assess model accuracy. The result is an average SHAP value for a given input to output association that captures the overall significance of a given input feature for a given output prediction.

### Current study

The current study had three primary aims: (1) investigate the use of SML trained LSTM-layered ANN models (hereafter referred to LSTM_*NN*_ models) to predict human decision-making during skillful joint-action; (2) demonstrate how SHAP can be employed to uncover the task information that supports human decision-making during skillful joint-action by determining what input information (features) most influenced the output predictions of a trained LSTM_*NN*_ model; and (3) apply these techniques to explicate differences between the decision-making process of novice and expert players while playing a simulated, fast paced herding game^67–69^.

Herding tasks involve the interaction of two sets of autonomous agents—one or more *herder* agents are required to corral and contain a set of heterogeneous *target* agents. Such activities are ubiquitous in daily life and provide a prototypical example of everyday skillful joint- or multiagent behavior. Indeed, while the most obvious examples involve farmers herding sheep or cattle, similar task dynamics define teachers corralling a group of young children through a museum or firefighters evacuating a crowd of people from a building^70^.

**Figure 1:**
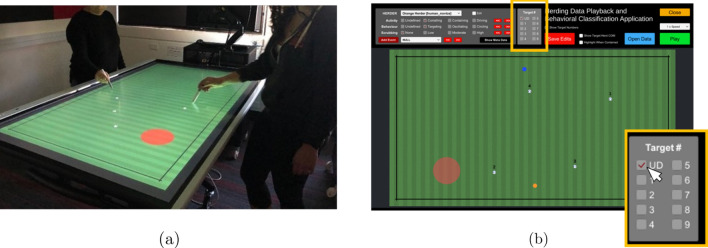
(**a**) The herding task and experimental setup. (**b**) A screenshot of the data playback and coding application used to identify which target a herder was corralling at each time step. The blue and orange dots are the herders and the white dots are the targets. The text label above each target specifies the target number (i.e., 1 to 4). The transparent red area is the containment region, which for the data employed here was always in the center of the game field. The insert panel on the bottom right of (**b**) is the target coding panel, where UD = no target. See main text for more details.

For the current study, we modeled data from^71^, in which pairs of players controlled virtual herder agents to corral a herd of four virtual cows (hereafter refereed to as *targets*), dispersed around a game field, into a red containment area positioned at the center of the game field. The task was presented on a large touch screen, with players using a touch-pen stylus to control their virtual herders (Fig. 1a). Targets were repelled away from the human-controlled herders when the herder came within a certain distance of the target. When not influenced by a herder, the movement of targets was governed by Brownian motion, with targets randomly (diffusely) wandering around the game field.

To successfully corral the targets into the containment area, previous research has demonstrated that effective task performance requires that players coordinate their target selection decisions by dynamically dividing the task space into two regions of responsibility, with each player then corralling one target at a time in their corresponding regions of responsibility until all of the targets are corralled within the containment area^68,71^. To date, however, no previous research has explicitly explored or modeled the process by which players make their target selection decisions, nor what state information regulates this decision-making behavior. Assuming that a player’s (a) target selection decisions are specified in the movements^72–74^ of their herder and (b) that players used kinematic information of target and herder states (e.g., relative positions, velocities) to make target selection decisions^20–22,68^, we expected that an LSTM_*NN*_ could be trained via SML to predict these target selection decisions using short sequences of herder and target state input features that preceded target selection. We expected that target selection decisions could be predicted prior to a player enacting a target selection decision and, given that experts perform significantly better than novices (^71^ and Supplementary Information, Sec. 1), we also expected that the trained LSTM_*NN,expert*_ and LSTM_*NN,novice*_ models would be expertise specific. Finally, we expected that SHAP could be employed to identify differences in the decision-making processes of the expert and novice herders, or more specifically, how accurate LSTM_*NN*_ models of expert or novice target selection behavior deferentially weighted task information (feature inputs) when making predictions.

## Results

Given players could choose to corral no target, predicting the target selection decisions of human herders corresponded to a 5-label prediction problem, with ID = 1, 2, 3, 4 corresponding to the actual targets and ID = 0 corresponding to no target.

To train an LSTM_*NN*_ using SML, we extracted time-series data from the data recordings of the expert-expert pairs and the successful novice-novice pairs that completed the dyadic herding task detailed in^71^. Only data from successful trials was employed. For each trial, state data was extracted from the time of task onset to when the players had corralled all four targets inside the containment area. At each time step, the target a herder was corralling was labeled manually by a research assistant, blind to the studies true purpose, using a custom coding software package (see Fig. 1b and “Methods” for more details). From the resulting labeled time-series data, training samples that included 48 input features were constructed of length *t*_*i*_ to *t*_*f*_, where *t*_*f*_ − *t*_*i*_ = *T*_*seq*_ and the length of *T*_*seq*_ corresponded to 25 time steps (i.e., *t*_*i*_ = *t*_*f*_ − 25 time steps) or 1 s of system state evolution. The 48 state input features were the *relative radial and angular distance* between herders and between the herders and each of the 4 targets, each herder’s and target’s *radial and angular distance from the containment area*, and the *radial velocity*, *radial acceleration* and *direction of motion* of each herder and target.

### Predicting future target selection decisions

LSTM_*NN*_ models were trained to predict the next target ID a herder would corral at *t*_*f*+*Thor*_ given a feature input sequence of length *T*_*seq*_, with *T*_*hor*_ > 0 time steps. Note that *T*_*hor*_ = 1 corresponded to predicting the target the herder would corral at the next time-step (equivalent to 40 ms in the future) and, thus, simply entailed predicting target selection decisions already made and/or currently being enacted by a herder. Here we present the results for models trained to predict target selection decisions at two longer prediction horizons, namely, *T*_*hor*_ = 16 and 32, which corresponded to predicting the target a herder would corral 640 ms and 1280 ms in the future, respectively (for comparative purposes, we also trained models for *T*_*hor*_ = 1 and 8, see Supplementary Information, Sec. 3).

Importantly, *T*_*hor*_ = 16 and 32 involved predicting the target selection decisions before a player’s decision or behavioral intention was enacted or typically observable in the input data sequence *T*_*seq*_. This was validated by calculating the average time it took players to move between targets when switching targets, with an average inter-target movement time of 556 ms for novice herders and 470 ms for expert herders (see Supplementary Information, Sec. 2).

**Figure 2:**
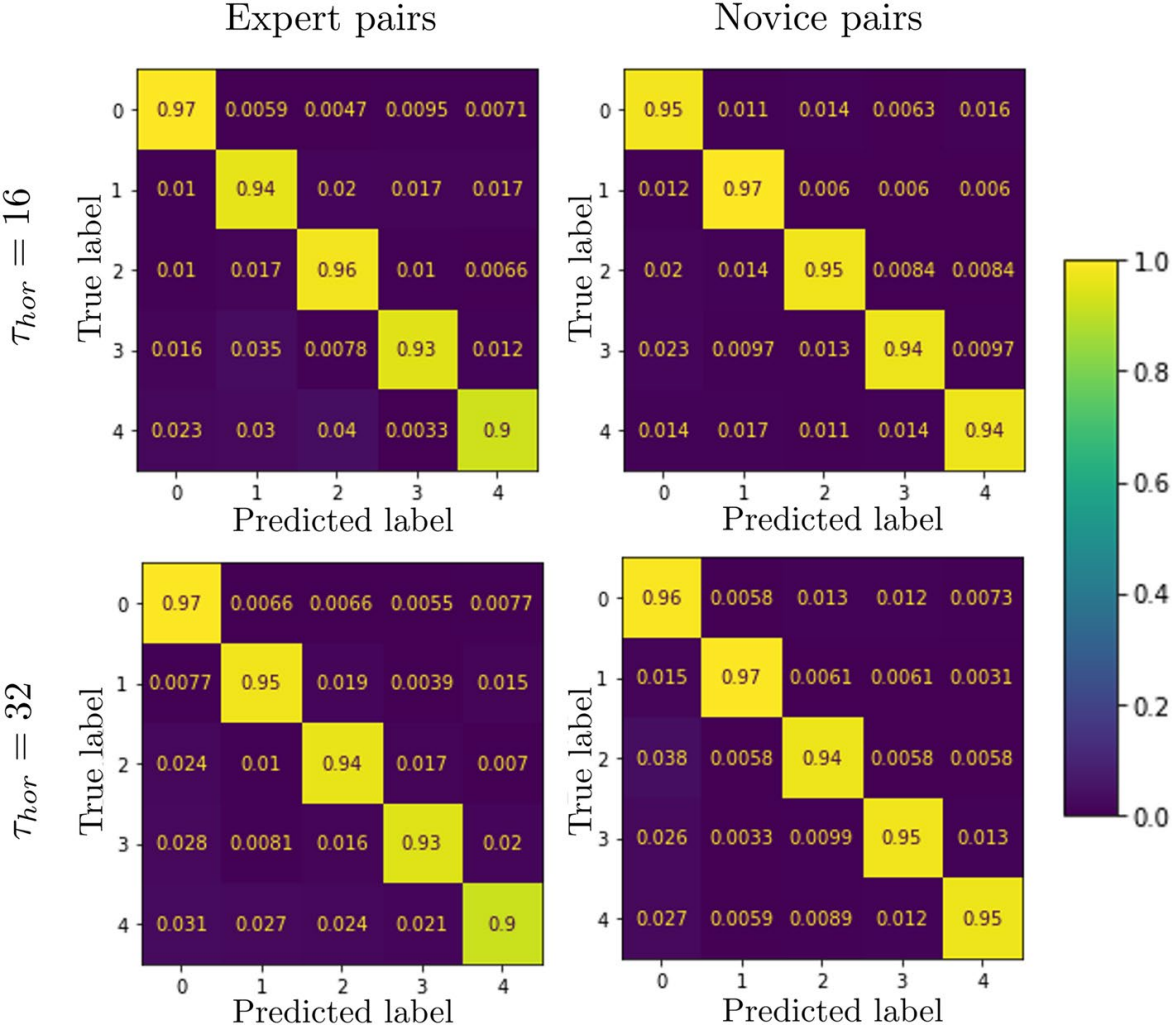
Confusion matrices for the trained prediction models tested on 10 sets of N_test_ = 2000 samples for each expertise level and prediction horizon. Values on the diagonal indicate the portion of test samples correctly predicted.

Separate LSTM_*NN*_ were trained to predict the target selection decisions of novice and expert herders for each prediction horizon. The confusion matrices of each LSTM_*NN*_ evaluated on ten sets of test data samples (i.e., samples not employed during training) are reported in Fig. 2. Importantly, the values on the diagonal indicate that each target ID could be correctly predicted between 90 and 97% of the time. Indeed, independent from prediction horizon and player expertise, each LSTM_*NN*_ predicted which target a herder would corral with an average accuracy exceeding 94% (see Table 1 for more prediction metrics, defined in “Methods”).

**Table 1:** Average performance [%] of the expert and novel models as a function of prediction horizon, tested on 10 sets of N_test_ = 2000 samples.

	Accuracy	Precision	Recall	F1 score	MI
*τ*_*hor*_ = 16 (640 ms) prediction horizon					
Novice	95.33 ± 0.2	95.18 ± 0.2	95.25 ± 0.2	95.2 ± 0.2	84.16 ± 0.5
Expert	95.2 ± 0.4	94.17 ± 0.6	94.5 ± 0.5	94.3 ± 0.5	84.9 ± 0.5
*τ*_*hor*_ = 32 (1280 ms) prediction horizon					
Novice	95.75 ± 0.5	95.42 ± 0.5	95.5 ± 0.5	95.45 ± 0.5	83.24 ± 1.04
Expert	94.66 ± 0.5	93.2 ± 0.7	93.34 ± 0.8	93.25 ± 0.7	81.32 ± 1.5

*MI* Mutual Information.

### Predicting differing target selection behaviors

**Figure 3:**
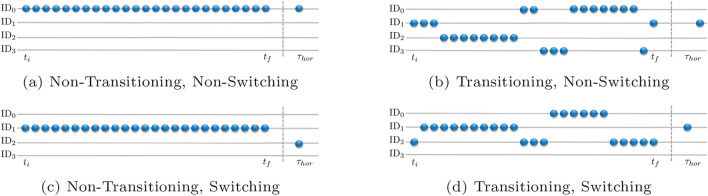
Illustration of the different sub-categories of target selection behavior, which also reflects the four different types of data samples used for model training and testing. Non-transitioning (**a**, **c**) and transitioning (**b**, **d**) corresponded to whether a harder corralled the same target or different targets during the input sequenced *T*_*seq*_ = *t* ∈ [*t*_*i*_*,t*_*f*_]. Non-switching (**a**, **b**) and switching (**c**, **d**) corresponded to whether a herder was corralling the same or a different target, respectively, at *T*_*hor*_ and *t*_*f*_.

Recall that the data samples used to make a target selection prediction are vector time-series of the herding system’s state evolution for *t* ∈ [*t*_*i*_*,t*_*f*_], where *t*_*f*_ − *t*_*i*_ = *T*_*seq*_, and the prediction outputs are chosen as the ID of the target that will be corralled at *t*_*f*+*Thor*_ with *T*_*hor*_ 6 = 0. It is important to appreciate that during the time interval *T*_*seq*_, a human herder could either continuously corral the same target agent or *transition* between different targets. Here we classified these as *non-transitioning* and *transitioning* behavioral sequences, respectively. Furthermore, at *T*_*hor*_, a herder could be corralling the same target that was being corralled at the end of *T*_*seq*_ or could *switch* to a different target. Here we classified these two possibilities as *non-switching* and *switching* behaviors, respectively. Taken together, this defines four different sub-categories (subclasses) of target selection behavior (or data sample type), which are illustrated in Fig. 3.

**Figure 4:**
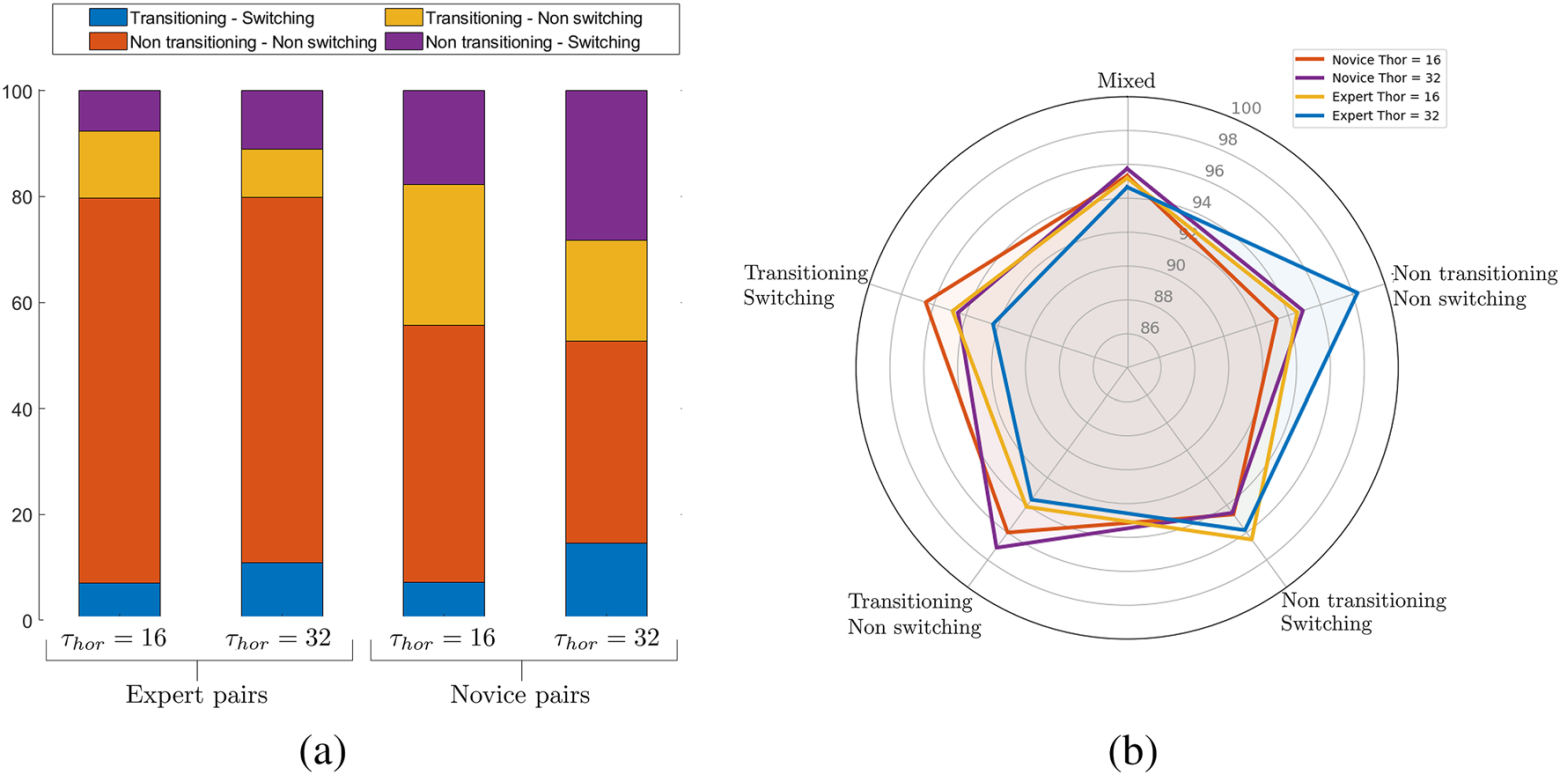
(**a**) Distribution of the different sub-categories of target selection (sample type) as a function of expertise level and prediction horizon. (**b**) The general (mixed) and sample specific accuracy of models trained using a uniform distribution of training samples (i.e., training set contained 25% of each sample type) as a function of expertise level and prediction horizon. Accuracy values ranged from 92.3 to 98.2%, with an overall mean accuracy of 95.23% while Mutual Information ranged from 66.6 to 93.5%, with an overall MI score of 81.5%.

The sample distribution of the different target selection behaviors observed within the expert and novice data set as a function of prediction horizon, *T*_*hor*_ = 16 and 32, are displayed in Fig. 4a. Interestingly, the expert data contained a high proportion of non-transitioning-non-switching behavior (69%), whereas the novice dataset contained a more even distribution of sample type compared to experts, particular for *T*_*hor*_ = 32. This indicated that experts both transitioned and switched between different targets less often than novices and were more persistent in corralling a given target compared to novices. Of more significance with regard to target selection predictions was that differences in sample type distribution could skew model accuracy due to an uneven representation of each sample type during training. That is, models trained using randomly selected training sets would exhibit lower accuracy for the underrepresented behavior types; e.g., model accuracy would be lower for transitioning-switching behaviors compared to non-transitioning-non-switching behaviors for example. This is illustrated in Supplementary Information, Sec. 4, where we show how model accuracy is sub-class dependent when models are trained using representative distributions of sample type.

Accordingly, the LSTM_*NN*_ models reported here were trained using training sets that contained a randomly selected, but uniform (balanced) distribution of sample type, such that each sub-class of target selection behavior was equally represented during training. Furthermore, in addition to examining overall model accuracy as a function of target ID (see Fig. 2 and Table 1), the LSTM_*NN*_ models predicting expert and novice target selection decisions at *T*_*hor*_ = 16 and 32 were also tested against *N* ≥ 1 novel test sets composed of either (i) non-transitioning-non-switching, (ii) non-transitioning-switching, (iii) transitioning-non-switching, (iv) transitioning-switching samples. Note that each test set contained 2000 test samples and thus the number of test sets *N* employed for model testing varied from 1 to 10 and was a function of how many samples were available post training (i.e., from the set of samples not employed during model training); see “Methods” for more details.

Average model accuracy for each sample type as a function of expertise level and prediction horizon is illustrated in Fig. 4; for a detailed list of model accuracy and mutual information values as a function of sample type see Supplementary Information, Sec. 4. The results revealed that the accuracy of the resultant models were essentially sample type independent. More specifically, for *T*_*hor*_ = 16 the accuracy for each sample type ranged between 93.3% and 98.2% for LSTM_*novice*_ models and 93.6% and 97.11% for LSTM_*expert*_ models, with an average mixed sample type accuracy of 95.33% (± 0.2) and 95.2% (± 0.4) for LSTM_*novice*_ and LSTM_*expert*_ models, respectively. Similarly, for *T*_*hor*_ = 32 the accuracy for each sample type ranged between 94.56% and 96.51% for LSTM_*novice*_ models and 92.32% and 96.5% for LSTM_*expert*_ models, with an average mixed sample type accuracy of 95.75% (± 0.5) and 94.66% (± 0.5) for LSTM_*novice*_ and LSTM_*expert*_ models, respectively.

### Specificity of expert and novice predictions

**Figure 5:**
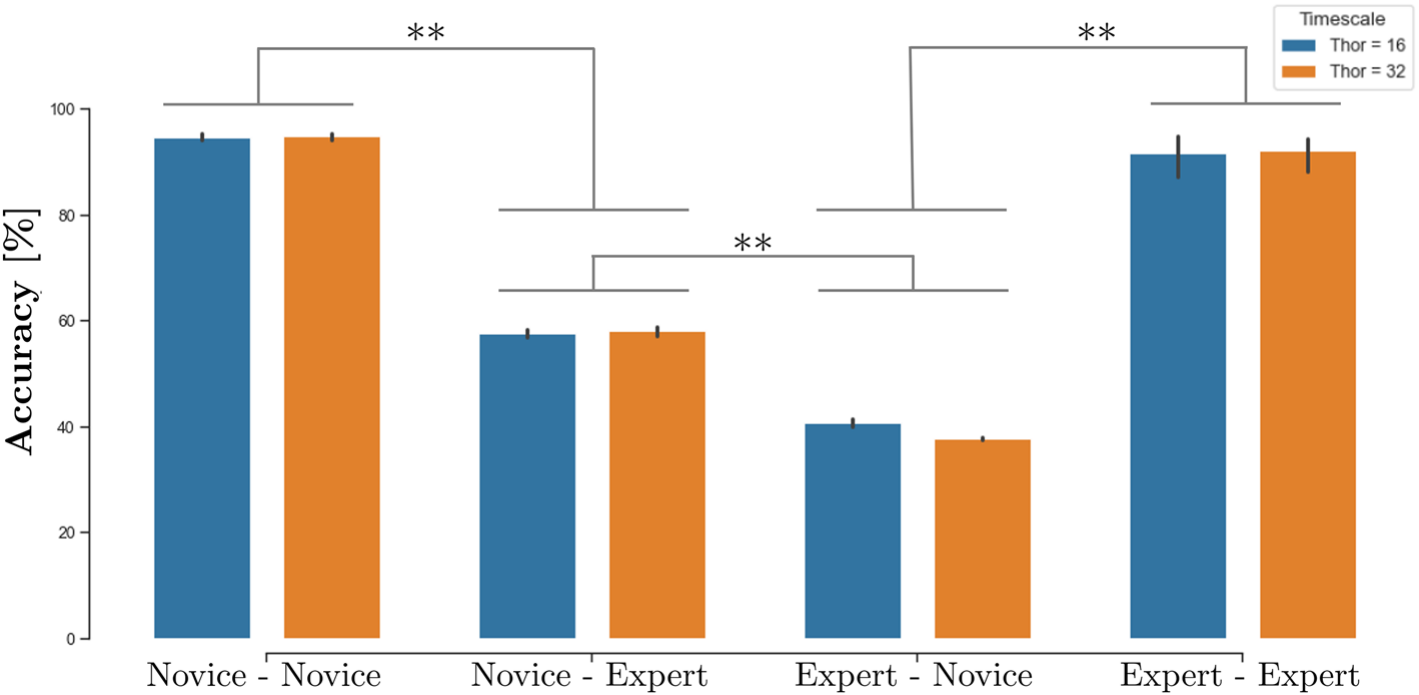
Average accuracy (%) of expert and novel trained models for 12 different tests sets of congruent (novice-novice, expert-expert) or in-congruent (novice-expert, expert-novice) *N*_*test*_ = 2000 samples. **Indicates a significant paired samples t-test difference of *p* < 0.01. Note there were no significant differences between the accuracy of LSTM_*novice*_ and LSTM_*expert*_ models for both prediction horizon’s when tested on the same expertise level (i.e., novice-novice and expert-expert (all *p* > 0.1).

The latter results provided clear evidence that the LSTM_*NN*_ models could accurately predict the future target selection decisions of expert and novice herders, independent of whether the future target to be corralled was the same or different from that being corralled at *T*_*hor*_ ≤ 0. With regard to differentiating expert and novice performance, of equal importance was determining whether the corresponding LSTM_*NN*_ models were expertise specific. This was tested by comparing the performance of the expert trained LSTM_*NN*_ models attempting to predict novice target selection decisions and vice versa. As expected, when an LSTM_*NN*_ trained on one expertise was used to predict test samples extracted from the opposite expertise model performance decreased to near or below chance levels (see Fig. 5), confirming that the models were indeed expertise specific. More specifically, for *T*_*hor*_ = 16 the LSTM_*expert*_ models predicted novice samples with an average accuracy of only 40.68%. Similarly, the LSTM_*novice*_ models only predicted expert samples with a 57.7% average accuracy. For *T*_*hor*_ = 32, average accuracy dropped to 37.66% for expert-to-novice and to 58.1% for novice-to-expert predictions.

### Identifying differences in expert and novice target selection decisions

The significant difference in the performance of LSTM_*NN*_ models trained and tested on the same level of expertise (see Table 1) compared to different levels of expertise (see Fig. 5) implied that the novice and expert LSTM_*NN*_ models weighted input state variables differently. Recall that, of particular interest here was whether these differences could be uncovered using the explainableAI technique SHAP. Accordingly, for each model we computed SHAP values for each sample in a in a subset of the training set and then, using the average SHAP value, rank-ordered each input feature in terms of its predictive importance.

Before assessing what specific input features were weighted differently, we first computed the ordinal association of SHAP value rankings between the different LSTM_*NN*_ models using the Kendall rank correlation coefficient (Kendall’s *τ*^75^) where *τ* = 0 corresponds to the absence of an association.

Note that although *τ* = 0 is the null-hypothesis and one cannot draw conclusions from non-significant results, it does provide a robust and intuitive assessment of rank order independence, *τ* = 1 corresponds to perfect association (matched rankings), and *τ* =  − 1 corresponds to opposite ranking orders (negative association). More specifically, we computed Kendall’s *τ* on the SHAP rankings of the full input feature set and on the top 10 features ranked by SHAP, between the novice and expert LSTM_*NN*_ models for each prediction horizon. Consistent with the expectation that novices and experts employed different state information when making target selection decisions, this analysis revealed little association between the novice and expert SHAP rankings for both *T*_*hor*_ = 16 and 32, with an average *τ* = 0.08 (*p* > 0.45) for *T*_*hor*_ = 16 and *τ =  − 0.09*, (*p* > 0.4) for *T*_*hor*_ = 32 (see Supplementary Information, Sec. 5 for a detailed summary of Kendall’s *τ* values).

**Figure 6:**
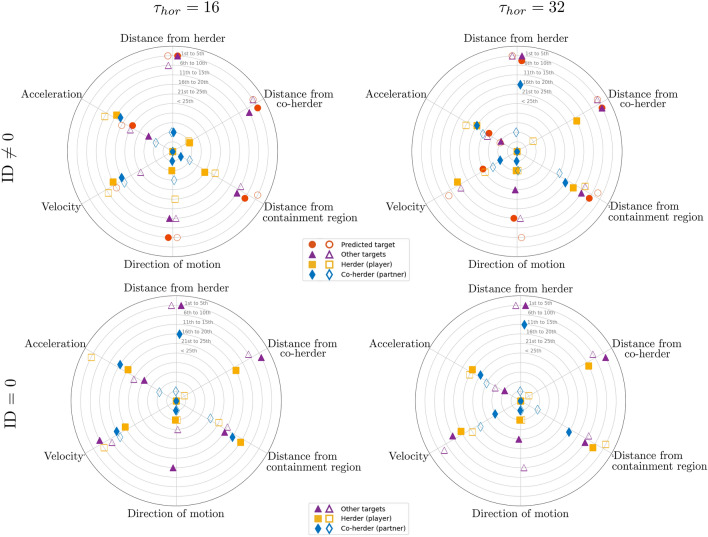
SHAP results for target prediction (*top row*) ID 6 = 0 (i.e., ID = 1 to 4) and (*bottom row*) ID = 0, as a function of prediction horizon and expertise (novice = empty shapes, expert = filled shapes). Feature type is listed on the vertices. The radial axis represents the average rank position, such that “1st to 5th” represents a feature that was always or nearly always ranked as a top-five feature.

To highlight what input features most influenced target selection predictions, Fig. 6 illustrates the average ranking of the different input features for both non-zero prediction outputs (i.e., ID = 1 to 4) and for ID = 0 prediction outputs, with the different input features defined as a function of input feature class (e.g., distance from herder, distance from co-herder, distance from containment area, velocity, etc.) and agent type. Note that the player that the target prediction corresponds to is referred to as the *herder*, a player’s partner is labeled as *co-herder*, the *predicted target* is the target that was predicted to be corralled by the herder at *T*_*hor*_ and *other-targets* corresponds to the targets that were not predicted to be corralled at *T*_*hor*_ (see Supplementary Information, Sec. 6 for a detailed summary of SHAP feature values).

Note that we split the illustration and analysis between ID 6 = 0 and ID = 0, as there is a qualitative difference between deciding to pick a specific target to corral and choosing not to corral a target, with the latter corresponding to either a period of indecision, a period of inter-target switching, or a decision that no target needed to be collared at that time. It is also important to appreciate that there is a non-trivial difference in how one should interpret the SHAP results as a function of prediction horizon. Although accurately predicting the target selection decisions of herders further in the future provides compelling evidence of the predictive power of SML based LSTM_*NN*_ modeling (and the potential stability or predictability of human behavior), predicting these decisions well in advance of the time that decision is made can provide less insight about what information the herder actually used. As noted above, the opposite is also true, for small prediction horizons the mapping between input features and model predictions might also provide less insight about what information a herder used to make a target selection decision, as the herder is likely already enacting a made decision.

For the present work, we continued to focus on *T*_*hor*_ = 16 and 32 (i.e., 640 ms and 1280 ms, respectively), as these two prediction horizons appeared to provide a good approximation of the lower and upper bounds of the timescale of herder target selection decisions. This was again motivated by the analysis of the players inter-target movement time, which as detailed above was on average less than 600 ms for both experts and novices. More specifically, for experts, 75.16% of their inter-target movement times were less than 640 ms, with 97.38% below 1280 ms. Similarly, for novices, 69.54% of their inter-target movement times were less than 640 ms, and 91.36% below 1280 ms (see Supplementary Information, Sec. 3). Thus, given the fast-paced nature of the task and the assumption that herders typically decided which target to corral next just prior to action onset^68,71^, it seemed likely that the majority of herder target selection decisions were made within this 640 to 1280 ms window.

With regard to target ID 6 = 0 predictions, a close inspection of Fig. 6a revealed that the relative distance between the herder and the predicted target and the herder and the other (non-predicted) targets were consistently identified as the key input features for both expert and novice predictions. Indeed, these features nearly always ranked within the top 5 features on average, independent of prediction horizon. The distance of the predicted target from the containment region was also nearly always ranked as a top 10 feature independent of expertise and prediction horizon. Interestingly, these results are consistent with the heuristic target selection models previously developed by^69,71^, in which human herders completing the same herding task explored here were assumed to pick targets that were (a) furthest from the containment region, but (b) closer to themselves than to their co-herder. Thus, the current results provide empirical support for these previous assumptions.

Recall, that the results of the Kendall’s *τ* analysis found very little rankorder similarity between novices and experts. Given the latter SHAP results, this implied that although expert and novice decisions appeared to be founded on similar target distance information, the specific importance ordering of target distances as a function of feature class was different across experts and novices.

Indeed, a closer inspection of the top 10 feature rankings of experts and novices (see Supplementary Information, Sec. 6) revealed that for expert target selection predictions the distance of targets from the co-herder were always ranked as the most important feature, whereas for novices the distance of targets from themselves (i.e., the herder) were more often ranked as the most important feature. Although, this difference may seem subtle, it suggests that experts were more attuned to what targets better afforded corralling by their co-herder.

Further support for the latter conclusion was provided by the SHAP results for target ID = 0, where the distance of targets from a player’s co-herder were consistently ranked as a top 5 feature for experts, but not for novices; see Fig. 6b. That is, the decisions of experts to not corral a target appeared to be more evenly determined by the distance of targets from themselves (i.e., distance from herder) and their co-herder, whereas novices decisions were more heavily weight by the distance of targets from themselves. Again, this suggest that experts were more attuned to what target selection decisions were best actualized by their co-herder compared to novices.

Consistent with the recent results of^76^, the SHAP analysis revealed that information about the direction of motion of the predicted targets also played an important role in the target selection decisions of experts and novices. Another subtle difference between expert and novice predictions, however, was that this finding was more pronounced for experts compared to novices, with the direction of motion of the predicted target ranked as a top 10 feature on average for novices and as a top 20 feature on average for experts for both *T*_*hor*_ = 16 and 32. Direction of motion was also more heavily weighted overall for expert target ID = 0 predictions in the shortest prediction horizon. This implied that expert target selection decisions were more heavily influenced by whether a target was moving towards or away from the containment region. Indeed, key to task success is ensuring that targets are moving towards the containment region, irrespective of the distance from the containment area (given that targets are constrained to move within a defined “fenced” boundary). Thus, it is often better to choose to corral a closer target that is moving away from the containment region, than to choose a target that is further away but moving towards the containment region.

Finally, the other difference between the SHAP results for expert and novice predictions related to the importance of herder acceleration and velocity. Indeed, these features were often ranked as a top 15 feature for novice predictions, particularly for no-target predictions when *T*_*hor*_ = 16. Given that 29.54% of novice inter-target movement times were greater than 640 ms, this result may be due to the novice LSTM_*NN*_ models simply learning to map the movement information of novice herders to the periods of target ID = 0 that occur during inter-target movements. That is, rather than indicating that novices herders were influenced by their own velocity or acceleration, this may be a consequence of the slower action-decision timescales and inter-target movements of novices compared to experts. Finding that herder velocity were less important for novice predictions when *T*_*hor*_ = 32 is consistent with this possibility. Thus, returning to the question of what prediction horizon best captured the decision timescale of herders, the latter result suggests that the SHAP results for the *T*_*hor*_ = 32 prediction horizon may better reflect the information employed by novice herders when making target selection decisions.

## Discussion

The current study leveraged recent advances in SML based LSTM modeling and explainable AI methods to model and understand the decision-making activity of expert and non-expert human actors performing a complex, fast-paced multiagent herding task^68,77^. Results revealed that short (1 s) state information sequences (*T*_*seq*_) could be used to train LSTM_*NN*_ models to accurately predict the target selection decisions of both expert and novice players. Importantly, model predictions were made prospectively, with the majority of predictions for *T*_*hor*_ ≥ 16 occurring before the target selection decision of herders were enacted or observable within the state input sequence. It is important to note that model effectiveness was not restricted to *T*_*seq*_ = 1 s or *T*_*hor*_ ≥ 16. As detailed in the Supplementary Information, Sec. 3–4, LSTM_*NN*_ models trained using sequence lengths of 0.5 to 2 s could accurately predict (above 95%) target selection decision at prediction horizons ranging from 20 ms to 2.56 s. Moreover, although correct predictions at *T*_*hor*_ ≥ 16 does not provide definitive evidence that these predictions preceded a herders intent, this possibility seems likely as the action decisions made by human actors during skillful action are spontaneously tuned responses to the unfolding dynamics of a task^13,18,20^ and, for the type of perceptual-motor task investigated here, often only occur 150 to 300 ms prior to action onset^78^. A significant implication is that the current modeling approach could be employed for the anticipatory correction of human action decisions during task training and real-time task engagement, as well as to develop more ‘human-like’ artificial or robotic agents^79^ that are capable of robustly forecasting and reciprocally adjusting to the behavior of human co-actors during human–machine interaction contexts.

An interesting avenue for future research would be to explore the degree to which the current modeling approach could be employed to predict human decision-making events across a variable prediction horizon. For instance, for the current task context this would equate to predicting target switching decisions. Future research could also explore the functional relationship between prediction horizon length and accuracy as a function of the timescale of a task and its decision-making dynamics. Of interest would be whether the approach employed here can be adapted from the fast-paced decision timescales that were explored here to tasks that involve much slower decision timescales (e.g., tasks where the involved actions decisions are taken over tens of seconds or minutes).

A key finding of the current study was that the trained LSTM_*NN*_ models were expertise specific, in that, when the expertise level of the training and test data was mismatched, prediction performance dropped to near chance levels. Consistent with decisions of skillful actors being a function of an actor’s trained attunement to the information that best specifies what action possibilities will ensure task completion^20–22^, this resulted from the expert and novice LSTM_*NN*_ models weighting input features differently. These differences were identified using SHAP, with average SHAP feature rankings revealing that experts were more influenced by information about the state of their co-herders and were also more attuned to target direction of motion information compared to novices. Together with finding that experts transitioned between targets less often than novices, this suggests that experts were more attuned to information that better specified the collective state of the herding system, including when and what targets afforded corralling by themselves and their co-herder.

To our knowledge, no previous research has employed an explainable AI technique to try to understand and explain the decision-making behavior of human actors during skillful action, let alone identify the differences between expert and novice actors or experimental conditions within the context of coordinated joint-action. To date, research on explainable AI has focused on the ability of these techniques to make AI models more understandable to human users^80,81^ and to argument or enhance the decision making capabilities of human users^82^. And, while these work have often drawn connection to cognitive and psychologies models and theories of human decision making, the utility of explainable AI for specifically understanding the how, why and when of human decision making has not been considered (for an exception, see^83^). We openly acknowledge that employing explainable-AI and SML trained LSTM_*NN*_ to understand human decision-making is based on two fundamental assumptions: (i) that the input features employed for model training includes the informational variables employed by human actors and (ii) that the mapping between input feature weights and model predictions is isomorphic with the actual information-decision mapping that underlies human action decisions; and that these assumptions need to be validated in future work.

We also acknowledge that task explored here provided players (herders) with access to full (global) state information. That is, at any given time, the herders could (more or less) always see the positions and movements of the other herder and all the target agents. Accordingly, another interesting avenue of future research is to explore whether the SML and explainable-AI approach proposed here can also be employed to model and understand human decision making when full access to the state of the task environment or system is inaccessible. This could be addressed, for example, by attempting to model the target selection decisions of human players completing a first-person herding game (e.g.,^84^), where each herder only has local (first-person field of view) information about the state of the task environment at any point in time.

Future research should also compare the effectiveness of the SHAP technique employed here with other explainable AI tools, such as LIME^61^, Deep-Lift^62^ or LRP^63^, as well as interpretable Transformer techniques^85,86^. Future work could also explore the possibility of using explainable-AI to understand decision making in contexts where indecision often occurs^87^, as well as whether an explainable-AI analysis of the input–output mappings that underlie miss-classification or incorrect decision predictions could be used to understand ineffective decision-making.

Despite the need for future work, the current study provides initial evidence that explainable-AI techniques could provide a powerful tool for understanding the decision-making processes of human actors, including what information best supports optimal task performance. Moreover, although we limited the focused of the current study on informational variables relevant to a visual-motor coordination task, the approach proposed here could be employed across a wide array of task and informational settings (i.e., visual, auditory, haptic, linguistic, etc.). Thus, the potential implications for both basic scientific research and the applied development of decision-making assessment tools could be limitless.

## Methods

All methods and procedures employed for the study were in accordance with Macquarie University human research regulations and were approved by the Macquarie University ethics board (protocol 6457). Informed consent was obtained from all subjects that participated in the original data collection study (^71^, with this previous study covered under the same ethics protocol listed above).

### Human herding task and data

Novice and expert human performance data from the joint-action herding experiments conducted in^71^ were employed for the current study. The herding task (game), developed with Unity-3D game engine (Unity Technologies LTD, CA), required pairs ($\hat{N}_{H}$ = 2) of human participants (players) to control virtual herding agents to corral and contain $\hat{N}_{T}$ = 4 randomly moving target agents within a designated containment area positioned at the center of a game field. The task was performed on large 70″ touch screen monitors (see Fig. 1a), with the human participants using touch-pen stylus to control the location of motion of the herder agents. The targets’ movement dynamics were defined by Brownian motion when not being influenced by a herder agent, and when influenced by a herder agent would move in a direction away from the herder agent. During task performance, the position and velocity of all herders and targets (as well as other general game states) was recorded at 50 Hz. Pairs had a maximum of 2 min to corral the targets into the containment area, with task success achieved if pairs could keep the targets contained within the containment area for 20 s. Full details of the experimental set-up and data collection process can be found in^71^.

*Novice data* was extracted from 40 successful trials performed by 10 different novice pairs (4 successful trials from each pair). From each trial, we extracted state data from the time of task onset to when all four target agents were first contained within the specified containment area; that is, when the herders had corralled all the agents inside the containment area for the first time. The remaining trial data was disregarded as players treat the target herd as single entity after it is contained and individual target selection decisions no longer occur^69,71^. Note that a human herder was considered to be a “novice” if they were unfamiliar with the herding task prior to the data collection session. Novices repeated the task until they had completed the 4 successful trials included in the novice data-set (with an average of 8 unsuccessful trials per pair).

*Expert Data* was extracted from 48 successful trials performed by 3 pairs of human players with extensive experience (completed more than 100 successful trials) performing the simulated multiagent herding task (i.e., several authors from^71^). As with the novice data, we extracted state data from the time of task onset to when all four target agents were first contained within the specified containment area.

### State input features

From position and velocity data recorded in the original novice and expert data-sets we extracted and derived the following *N*_*sv*_ = 48 state variables:

- The radial and angular distance (∆, Ψ) between herders,
- The radial and angular distance (∆_*i,j*_, Ψ_*i,j*_) of target *i* from herder *j*,
- The radial and angular distance of herder *j* or target agent *i* from the center of the containment region.
- The radial velocity and acceleration of herders ($\dot{r}\left( t \right), \, \ddot{r}\left( t \right)$) and target agents ($\dot{\rho }\left( t \right), \, \ddot{\rho }\left( t \right)$),
- The direction of motion of herder and target agents.

### Target coding

A paid research assistant, naive to the study’s purpose, coded (classified) what target (or not) a given herder was corralling at each point in time via an interactive data playback Unity3D (version 2018LTS) application that played back the original recorded data-set (see Fig. 1b). Data playback speed could be decreased to 1/8 speed, as well as stepped frame by frame, with each target visually labeled with a fixed number (1 to 4). At each time step, the target agent a given human herder was corralling was coded by the research assistant with an integer number $\tilde{i} \in \left[ {0,\hat{N}_{T} } \right]$ , with $\tilde{i}$ = 0 meaning “no target agent being corralled” and $\tilde{i}$ 6 = 0 being the class ID of the target agent being corralled at that time step.

### Human inter-target movement time

To determine the time it took expert and novice herders to move from one target to the next we calculated the inter-target movement time when switching between targets ID = 1 to 4 (i.e., we ignored switch events from or to ID = 0). We calculated the time from when a human herder moved outside the region of repulsive influence of the current target and entered the region of repulsive influence of the next target. More specifically, inter-target movement time was the difference in milliseconds between the time instant at which a herder entered the repulsive radius (i.e., 0.12 m around each target agent) of the current target and their relative distance was decreasing, and the time instant at which the herder left the repulsive region around the previously corralled target and their relative distance increased. In addition to the mean results report above, see Supplementary Information, Sec. 2 for the distributions of inter-target movement times for expert and novice herders.

### Training and test set data

All successful trial data, per level of expertise, was stacked in a common *feature processed* novice or expert data-set along with the corresponding target codes (ID 0 to 4). From the resultant, feature processed, novice and expert data-sets we randomly extracted 2 sets of *N*_*train*_ = 21,000 training samples and 20 test sets of *N*_*test*_ = 2000 samples each. The corresponding pseudo-code is reported in Algorithm 1.

The first training set and 10 test sets contained transitioning/switching samples in balanced proportion (25% each). This data set was used to train and test the models presented here. The second training set and the remaining 10 test sets contained transitioning/switching samples in the same proportion as in the entire data-set see Supplementary Information, Sec. 3–5 for information about the models trained using the latter unbalanced training sets.
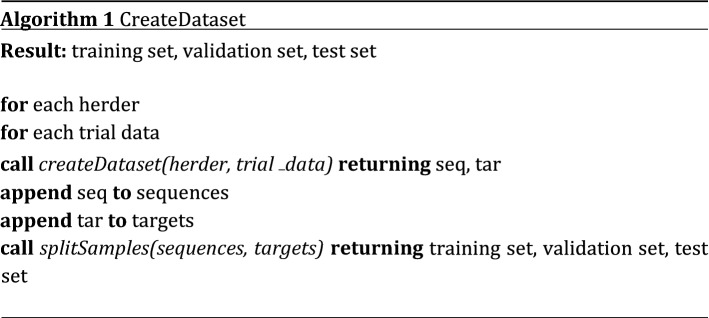


Here, samples refer to pairs of state feature sequences and target label codes. Sequences are composed by *N*_*seq*_ = 25 consecutive instances of the above listed *N*_*sv*_ state variables, sampled at *dt* = 0*.*04 s, covering *T*_*seq*_ = 1 s system evolution, and labels are the ID of the agent being targeted, selected at *T*_*hor*_ = 16*dt* and *T*_*hor*_ = 32*dt* seconds from the end of the corresponding sequence.

In Supplementary Information, Sec. 3 we also consider values of *T*_*seq*_ = 0*.*5 s and *T*_*seq*_ = 2 s varying the sampling time to *dt* = 0*.*02 and *dt* = 0*.*08 s respectively. As in the default case presented in the *Results*, the novice and expert models trained with these different *T*_*seq*_ lengths also obtain accuracy values greater than 95% when tested on data from the same expertise level (e.g., expert-expert) and closer to 50% when tested on data from the different level of expertise (e.g., novice-expert).

### LSTM network and model training

For each combination of expertise and prediction horizon, we trained a LongShort Term Memory (LSTM) artificial neural network with Dropout layers^44,45^, using Adam optimization and stratified K-fold cross-validation with K = 5 (code available at https://github.com/FabLtt/ExplainedDecisions). We used Bayesian Optimization to tune the learning rate (*α* = 0*.*0018) of the Adam optimizer and the hyperparameters of the LSTM_*NN*_ (i.e., the number of LSTM hidden layers, number of neurons in each layer, and dropout rates). The *Input Layer* and the output *Dense* layer of the optimized LSTM_*NN*_ had dimensionality (*T*_*seq*_, *N*_*sv*_) and (*T*_*seq*_, $\hat{N}_{T}$ + 1), respectively. In the center, 3 hidden LSTM layers of 253, 45 and 8 neurons were alternated with Dropout layers of equal dimensionality. The dropout rate of each LSTM layers of the novice and expert models was 0.1145. For the dropout layers between each LSTM layer, the dropout rate was 0.0145. To avoid over-fitting, training was stopped when the logarithmic loss—that penalises false predictions—on the validation set stopped improving; the validation set being a randomly extracted 10% of the training set. The LSTM_*NN*_ was built and trained using Python 3.7.1 and Tensorflow library (https://www.tensorflow.org/, version 1.15). The corresponding pseudo-code is reported in Algorithm 2.
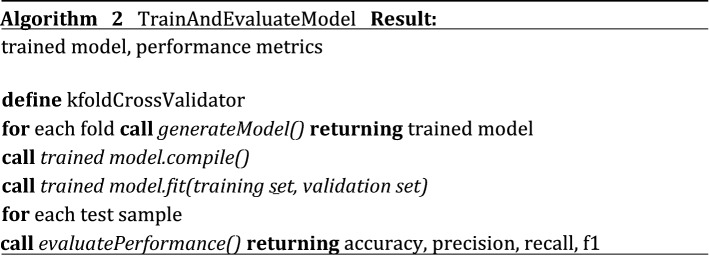


### Model performance

Performance of the LSTM_*NN*_ were validated using the following measures: *Accuracy*—the fraction of correct predictions outputs among the samples tested; *Precision*—how valid the prediction was, that is the portion of relevant outputs among the predicted ones; *Recall*—how complete the prediction was, that is, the portion of relevant outputs that were predicted. Note that when Precision and Recall reach 100%, false positive outputs and false negative outputs are absent, respectively. Additionally, we also report the *F1 score* for model prediction’s, with higher values of *F1 score*, the harmonic mean of Precision and Recall, expressing how precise and robust the model prediction was.

### Shapley additive explanation

Given a sample, the SHAP algorithm assigns to each input feature an importance value (https://github.com/slundberg/shap, version 0.31). This is an estimate of the contribution of each feature to the difference between the actual prediction output and the mean prediction output. We randomly selected $\hat{N}_{train}^{S}$ = 200 samples from the training set as background set, that is, as a prior distribution of the input space used to approximate the conditional expectations of the SHAP values^65^. We applied SHAP DeepExplainer on *N*_*test*_ = 6000 of the test samples used to evaluate performance^65^ and obtained the corresponding SHAP values for each state variable. To derive the corresponding approximate global feature importance measure (shown in Fig. 6) we averaged over the test set, for each class of prediction output (i.e., target ID). The corresponding pseudo-code is reported in Algorithm 3.
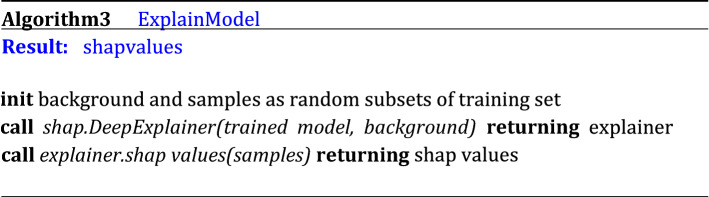


## Supplementary Information

Supplementary Information.

## Data Availability

The raw datasets and code employed for the current study are available at https://github.com/FabLtt/ExplainedDecisions. The processed datasets, models and detailed SHAP values are available at https://osf.io/wgk8e/?viewonly=8aec18499ed8457cb296032545963542.
