# Supplementary material for: Predicting and understanding human action decisions during skillful joint-action using supervised machine learning and explainable-AI
Source: Sci Rep. 2023 Mar 27;13:4992. doi: 10.1038/s41598-023-31807-1 (PMC10042997; doi:10.1038/s41598-023-31807-1)
Supplement: Supplementary file 1 — Supplementary Information. [file 41598_2023_31807_MOESM1_ESM.pdf]

Supplementary Information for "Predicting and Understanding Human Action Decisions during Skillful Joint-Action using Supervised Machine Learning and Explainable-AI"

Fabrizia Auletta[1,2], Rachel W. Kallen[1,3], Mario di Bernardo[4,5] and *Michael J. Richardson[1,3]

[1]School of Psychological Sciences, Faculty of Medicine, Health and Human Sciences, Macquarie University, Sydney, NSW, Australia.
[2]Department of Engineering Mathematics, University of Bristol, Bristol, United Kingdom.
[3]Center for Elite Performance, Expertise and Training, Macquarie University, Sydney, NSW, Australia.
[4]Department of Electrical Engineering and Information Technology, University of Naples, Federico II, Naples, Italy.
[5]Scuola Superiore Meridionale, Naples, Italy.

1 Expert and Novice Herding performance

As in [1, 2], the herding performance of players was assessed using the following five measures. (1) *Gathering time*, which is the time period $t_{\mathrm{g}} \in [0, T]$, where all the passive agents are within the containment area for the first time. (2) *Distance traveled by the herders* d_{g}, which is the mean distance (in meters) traveled by the herders during the time interval $[0, t_{\mathrm{g}}]$. (3) *Herd distance from containment region* D_{g}, which captures the herders ability to keep the herd close to the containment area, calculated with respect to the center of the containment area. A smaller average distance indicates better ability of the herders to keep the herd close to the containment region. (4) *Herd spread* S_{g}, which measures the scatter of the herd within the game field during the time interval $[0, t_{\mathrm{g}}]$. Lower values corresponds to a more cohesive herd and consequently better herding performance. The herd spread is evaluated with

respect to the area of the containment region, $A_{\rm cr} = \pi(r^\star)^2$, as $S_{g,\%} = S_{\rm g}/A_{\rm cr} \cdot 100$. And, (5) *Containment rate* $I_{\%}$, which measures the herders' ability to relocate one or more target agents inside the containment region. It is defined as the mean in time of the percentage of agents in the containment area during the time interval $[0, t_{\rm g}]$.

Table 1 Average performance of novice and expert pairs

	Novice pairs	Expert pairs
$t_{\rm g}$ [a.u.]	26.35±8.67	10.65±3.43
$d_{\rm g}$ [a.u.]	7.19±4.08	4.6±1.6
$D_{\rm g}$ [a.u.]	0.99±0.2	0.35±0.22
$S_{g,\%}$ [%]	3.61±1.99	2.7±1.48
$I_{g,\%}$ [%]	18.25±7.18	16.81±6.76

Performance was assessed with respect to the 48 expert and 40 novice data trials employed for model training and testing. The average and SD for each measure as a function of expertise is reported in Table 1, with experts performing better than novices with regard to all measures. More specifically, Kruskal Wallis statistical tests revealed significant differences between Novice and Expert pairs for gathering time $t_{\rm g}$ ($\chi^2 = 24.67$, $p < 0.0001$), distance traveled $d_{\rm g}$ ($\chi^2 = 5.76$, $p < 0.02$) and the average distance of the herd from the containment region $D_{\rm g}$ ($\chi^2 = 24.33$, $p < 0.0001$).

2 Inter-target movement times

For each successful trial, the inter-target movement times of experts and novice herders were determined by calculating the difference between the time a herder began influencing it's current target and the time the herder stopped influencing the previous target. Figure 1 reports the distribution of inter-target movement times for both expert and novice pairs. The average inter-target movement time was 556 ms for novices (with 65% of the total inter-target movement times $\leq 600ms$) and 470 ms for experts (with 72.5% of the total inter-target movement times $\leq 600ms$).

3 Performance of target selection models with different sequence lengths and prediction horizons

The SML approach presented in the main article can be customized to forecast the ID of the target that will be corralled by a herder for different lengths of state input sequence, T_{seq}, and different prediction horizons T_{hor}. Here, T_{seq} corresponded to a time-series of relevant state variables, fixed to $N_{seq} = 25$,

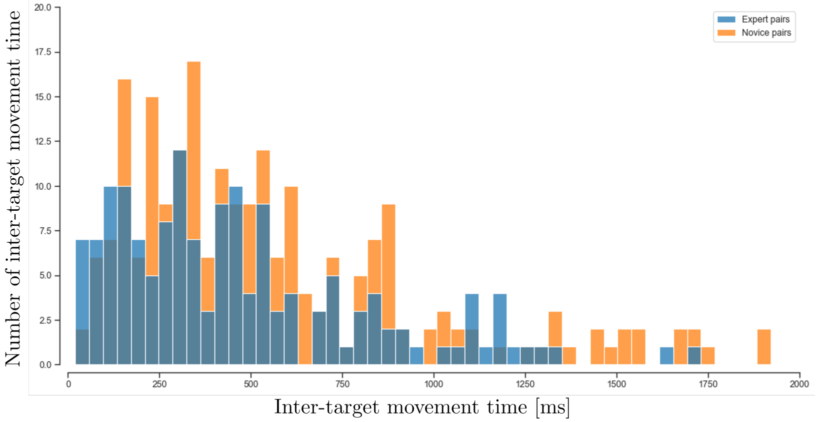

Fig. 1 Distribution of inter-target movement times [ms] of expert (blue) and novice (orange) herders. The average inter-target movement time was 556 ms (65% of the total inter-target movement times < 600 ms) for novices and 470 ms (72.5% of the total inter-target movement times< 600 ms) for experts.

such that T_{seq} is scaled by tuning the sampling time dt. The output prediction is the ID of the next target to be corralled by the herder at T_{hor} in the future.

In the main text we reported the accuracy of models trained using $T_{seq} = 1$ second of system state evolution (i.e., $dt = 2$ or 40 ms) and prediction horizons $T_{hor} = 16dt$ and $32dt$, which corresponded to prediction horizons of 640 ms and 1280 ms, respectively. However, we also trained models for $T_{seq} = .5$, and 2 seconds (where $dt = 1$ and 4 time steps or 20 and 80 ms, respectively) and for $T_{hor} = 1dt$ and $8dt$. Thus, T_{hor} ranging from 20 to 640 ms for $T_{seq} = .5$ seconds and from 80 ms to 2.56 seconds for $T_{seq} = 2$ seconds.

The accuracy values for the different combinations of T_{seq} and prediction horizon T_{hor} are reported in Figure 2. Overall, model accuracy was relative stable across the different combinations of T_{seq} and T_{hor} for both experts and novice models. Consistent with the results reported in the main text the models were also expertise specific for all combinations of T_{seq} and T_{hor}.

As mentioned in the main text, it is important to understand that when $T_{hor} < 600$ ms the prediction horizon entailed predicting a target selection decision that had already been made by a herder. Thus, for $dt = 1$ or 2 time steps, the $T_{hor} = 1dt$ and $8dt$ prediction horizons were of less interest here as the input data sequence would have included data from the enactment of the already made target selection decision (i.e., predictions were based on herder state information already specifying the made decision). They do, however, provide a benchmark measure of accuracy for the $T_{hor} = 640$ ms and 1280 ms models presented in the main text and for the $T_{seq} = 2$ second models for $T_{hor} \geq 8dt$. That is, $T_{hor} < 600$ ms predictions provide a measure of model accuracy when the target selection decision is potentially well specified in the input data (particularly for $T_{hor} = 1dt$). The fact that the model accuracy for $T_{hor} > 600$ ms was comparable to $T_{hor} << 600$ ms illustrates the robustness of

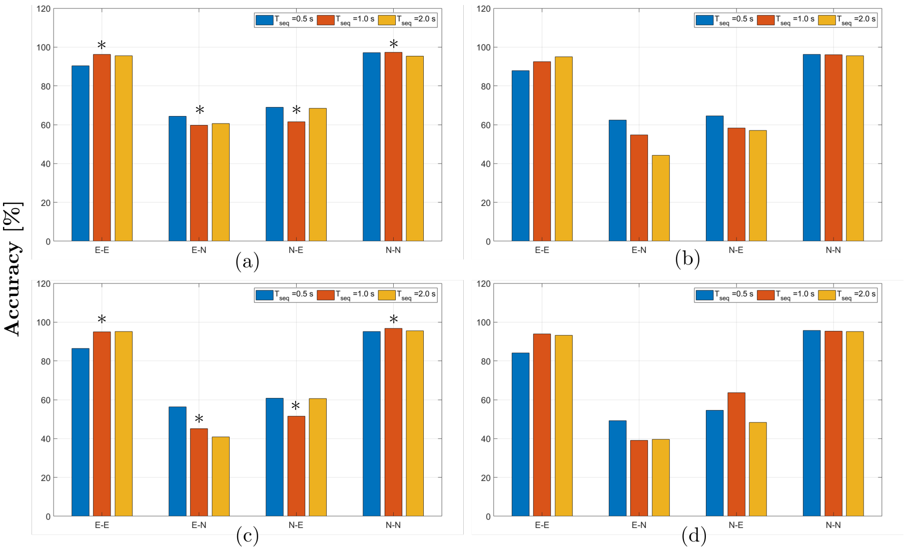

Fig. 2 Overall accuracy of LSTM$_{NN}$ models trained on $N_{test} = 2000$ unbalanced (representative) samples for different combination of training and test pairs (E = expert; N = novice) for (a) $T_{hor} = dt$, (b) $T_{hor} = 8dt$, (c) $T_{hor} = 16dt$, (d) $T_{hor} = 32dt$. $*$ indicates the accuracy values for the nominal case $T_{seq} = 1$ s reported in the manuscript.

the proposed SML-LSTM approach for predicting the target selection decisions both pre- and post-enactment.

4 Performance of target selection models with different type of samples

As detailed in the main text, during the time interval T_{seq}, a herder could either continuously corral the same target agent or transition between different targets. These were classified as "non-transitioning" and "transitioning" behavioral sequences, respectively. Similarly, at T_{hor}, a herder could be corralling the same target agent that was being corralled at the end of T_{seq} or "switch" to different target agent. These were classified as "non-switching" and "swithching" behavioral sequences, respectively. The resultant four data sample types are illustrated in Figure 3 in the main text.

Importantly, the number of "switching" samples within the data set used for model training and testing was dependent on T_{hor}. Indeed, both novice and expert sample data contained less than 2% transitioning-switching and less then 3% non-transitioning-switching samples when $T_{hor} = 1$, and less than 5% transitioning-switching and less than 7% non-transitioning-switching samples when $T_{hor} = 8$. The different distributions of sample type as a function of T_{hor} is illustrated in Figure 3.

Figure 4 details the performance of the trained LSTM$_{NN}$ models on $N_{test} = 2000$ test samples randomly extracted from the different sample types.

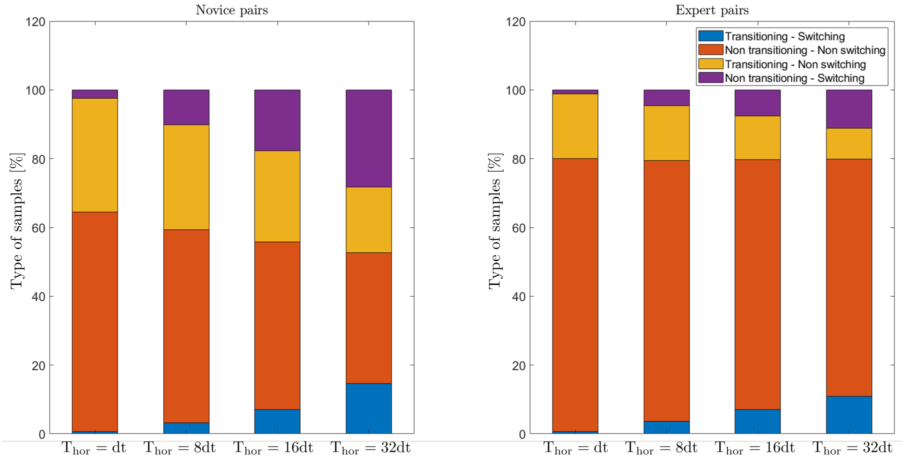

Fig. 3 Percentage different type of samples in the training set for different prediction horizon and $T_{seq} = 1$s.

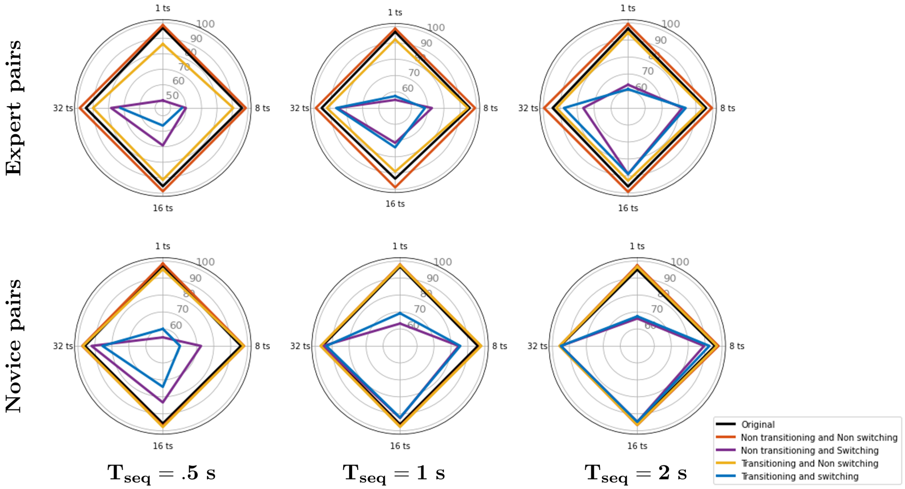

Fig. 4 Accuracy of models trained using unbalanced (representative) training sets, as as function of sequence length, prediction horizon, expertise and sample type. Vertexes are the accuracy of the trained models for each decision time interval T_{seq} and horizon T_{hor}. Accuracy for each model is scored on $\mathbf{N_{test}}$= 2000 samples of the corresponding sample type or the 'original" 2000 mixed samples).

That is, in contrast to the models presented in the main text, the accuracy values reported in Figure 4 reflect the accuracy of LSTM$_{NN}$ models trained on an unbalanced (representative) set of training samples. Not surprisingly, the accuracy of the models for a specific type of sample was dependent on the proportion of samples with the data set, with the accuracy for switching samples greatly reduced for $T_{hor} = 1dt, 8dt$ and $16dt$ (particularly when $T_{seq} = .5$ seconds; i.e., when $dt = 1$ or 20 ms), because of the reduced number of switching

Table 2 Sample type performance [% accuracy] for $T_{hor} = 16$ and $T_{hor} = 32$ models, when $T_{seq} = 1s$, trained using a representative (unbalanced) distribution of sample type. The % of each sample type with the data set is also reported. Models were tested on a corresponding sets of N_{test}= 2000 samples.

	Non transitioning				Transitioning				Mixed	
	Non switching		Switching		Non switching		Switching			
	Accuracy	% samples	Accuracy	% samples	Accuracy	% samples	Accuracy	% samples	Accuracy	% samples
$\tau_{hor} = 16$ (640 ms) prediction horizon										
Novice	97.35%	48.61%	92.48%	17.69%	97.69%	26.59%	92.22%	7.12%	96.21%	100%
Expert	98.86%	72.67%	71.45%	7.63%	89.14%	12.62%	74.24%	7.08%	93.48%	100%
$\tau_{hor} = 32$ (1280 ms) prediction horizon										
Novice	96.72%	38.14%	95.48%	28.27%	96.84%	19.02%	93.48%	14.57%	95.78%	100%
Expert	98.86%	69.06%	85.48%	11.11%	92.03%	9%	86.37%	10.84%	95.27%	100%

Table 3 Sample type performance [% accuracy] for $T_{hor} = 16$ and $T_{hor} = 32$ models, when $T_{seq} = 1s$, trained using a uniform (balanced) distribution of sample type (i.e, training set contained 25% of each sample type). Models were tested on a corresponding sets of N_{test}= 2000 samples.

	Non transitioning		Transitioning		Mixed
	Non-switching	Switching	Non-switching	Switching	
$\tau_{hor} = 16$ (640 ms) prediction horizon					
Novice	93.3	94.91	94.56	98.2	95.33±0.2
Expert	96±0.3	97.11±0.3	94.12±0.9	93.6±0.6	95.2±0.4
$\tau_{hor} = 32$ (1280 ms) prediction horizon					
Novice	94.68±0.8	94.56±0.4	96.51±0.6	95.83±0.3	95.75±0.5
Expert	96.5±0.5	94.5±0.5	94.83±0.2	92.32±0.73	94.66±0.5

samples in the training set. Indeed, there is a direct correspondence between the accuracy reported in Figure 4 and the proportion of a given sample type illustrated in Figure 3; also see Table 2. It is for this reason that the models reported in the main text were trained on balanced (uniform) data sets (i.e., training sets that included an equal number of each sample type) in order to ensure that model accuracy was sample type independent. Note, however, that balanced training was never possible for $T_{hor} = 1dt$, as there are never enough switching samples, even when $T_{seq} = 2$ seconds.

For comparative purposes, the accuracy of models trained using unbalanced (representative) and balanced (uniform) training sets for $T_{hor} = 16$ or 640 ms and $T_{hor} = 32$ or 1280 ms, when $T_{seq} = 1$ second, are detailed in Table 2 and Table 3, respectively. The accuracy values in Table 3 and Mutual Information in Table 4 correspond to those reported in the main text.

Table 4 S

ample type performance [% mutual information] for $T_{hor} = 16$ and $T_{hor} = 32$ models, when $T_{seq} = 1s$, trained using a uniform (balanced) distribution of sample type (i.e, training set contained 25% of each sample type). Models were tested on a corresponding sets of N_{test}= 2000 samples.

	Non transitioning		Transitioning		Mixed
	Non-switching	Switching	Non-switching	Switching	
$\tau_{hor} = 16$ (640 ms) prediction horizon					
Novice	77.86	83.89	82.81	93.54	84.16±0.5
Expert	66.56±2.05	89.88±0.77	81.36±2.28	80.44±1.5	83.24±1.04
$\tau_{hor} = 32$ (1280 ms) prediction horizon					
Novice	81.64±1.8	82.91±1.21	87.9±1.7	85.92±0.85	84.91±0.5
Expert	67±2.76	82.5±1.35	82.88±0.66	77.1±1.83	81.32±1.5

5 Kendall rank correlation coefficients

The ordinal association of SHAP value rankings (the first top 10 reported in Tables 6-6) was computed using the Kendall rank correlation coefficient (Kendall's τ) for subgroups of N top ranked input features. Table 6 includes the Kendall coefficients, and associated p-values, as a function of expertise. Table 5 includes the Kendall coefficients as a function of $T_{hor} = 16$ and $T_{hor} = 32$ prediction horizon for each level expertise.

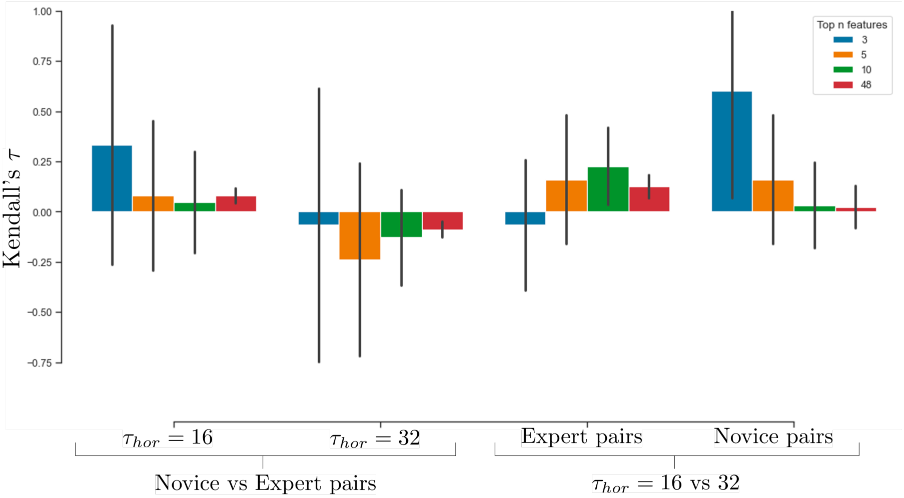

Fig. 5 K

endall's tau values for subgroups of top ranked input features averaged over labels for models trained on balanced data sets.

τ's values and corresponding p-values between novice and expert SHAP ranking for both $T_{hor} = 16$ and $T_{hor} = 32$ for models trained on balanced samples.

	$T_{hor} = 16$ (640 ms)					
	All features		Top 10		Top 5	
	Kendall tau	p-value	Kendall tau	p-value	Kendall tau	p-value
Label 0	0.078	0.43	0.34	0.22	0.4	0.48
Label 1	0.094	0.35	-0.07	0.86	0.0	1.0
Label 2	0.144	0.15	0.24	0.38	0.4	0.48
Label 3	0.060	0.54	-0.38	0.15	-0.6	0.23
Label 4	0.025	0.80	0.11	0.73	0.2	0.82
	$T_{hor} = 32$ (1280 ms)					
	All features		Top 10		Top 5	
	Kendall tau	p-value	Kendall tau	p-value	Kendall tau	p-value
Label 0	-0.03	0.76	0.02	1.0	-0.2	0.82
Label 1	-0.06	0.53	0.38	0.15	0.6	0.23
Label 2	0.05	0.63	0.06	0.86	0.4	0.48
Label 3	0.22	0.03	-0.02	1.0	0.2	0.82
Label 4	-0.06	0.56	-0.29	0.29	-0.2	0.82

Table 5 K

endall τ's values and corresponding p-values between $T_{hor} = 16$ and $T_{hor} = 32$ prediction horizons SHAP ranking for both novice and expert models trained on balanced samples.

	expert models					
	All features		Top 10		Top 5	
	Kendall tau	p-value	Kendall tau	p-value	Kendall tau	p-value
Label 0	0.07	0.45	0.24	0.38	0.2	0.82
Label 1	0.16	0.11	0.47	0.07	0.4	0.48
Label 2	0.04	0.68	-0.11	0.73	-0.2	0.82
Label 3	0.19	0.05	0.33	0.22	-0.2	0.82
Label 4	0.16	0.09	0.2	0.48	0.6	0.23
	novice models					
	All features		Top 10		Top 5	
	Kendall tau	p-value	Kendall tau	p-value	Kendall tau	p-value
Label 0	-0.11	0.28	-0.15	0.60	-0.8	0.08
Label 1	-0.05	0.61	0.11	0.73	0.6	0.23
Label 2	-0.03	0.76	-0.47	0.07	-0.6	0.23
Label 3	-0.11	0.25	0.15	0.60	-0.2	0.82
Label 4	-0.13	0.17	-0.29	0.29	-0.2	0.82

6 SHAP value tables for each feature for the target selection models

A detailed summary of SHAP feature values for each $LSTM_{NN}$ model, prediction horizon and target ID is provided in Tables 6-6.

10 ranked features and corresponding SHAP values for each class predicted by the model trained on *novice* pairs with a sequence $T_{seq} = 1$s and model trained on balanced data.

	$T_{hor} = 16$ (640 ms) prediction horizon									
	Label 0		Label 1		Label 2		Label 3		Label 4	
	Features	SHAP values	Features	SHAP values	Features	SHAP values	Features	SHAP values	Features	SHAP values
1	herd. accel.	0.028	herd. targ.0 dist.	0.028	herd. targ.1 dist.	0.025	herd. targ.2 dist.	0.024	herd.1 targ.3 dist.	0.042
2	herd.1 targ.3 dist.	0.027	herd.1 targ.0 dist.	0.024	herd.1 targ.1 dist.	0.024	herd.1 targ.2 dist.	0.020	herd. targ.3 dist.	0.031
3	herd. targ.0 dist.	0.026	targ.0 direction	0.017	targ.1 goal dist.	0.019	herd.1 targ.3 dist.	0.016	targ.3 goal dist.	0.020
4	herd. targ.3 dist.	0.025	targ.0 goal dist.	0.015	herd.1 targ.3 dist.	0.015	targ.2 goal dist.	0.015	herd. accel.	0.016
5	herd. targ.1 dist.	0.024	herd.1 targ.3 dist.	0.014	herd. targ.0 dist.	0.015	targ.2 direction	0.015	herd. targ.2 dist.	0.015
6	herd. velocity	0.023	herd. targ.1 dist.	0.014	herd. targ.3 dist.	0.015	herd. targ.3 dist.	0.013	herd. targ.1 dist.	0.015
7	herd. targ.2 dist.	0.022	herd. targ.3 rel angle	0.012	targ.1 direction	0.011	herd. targ.1 dist.	0.012	herd. targ.0 dist.	0.014
8	herd.1 targ.1 dist.	0.021	herd. targ.3 dist.	0.012	herd. targ.3 rel angle	0.011	herd. targ.0 dist.	0.012	herd. targ.3 rel angle	0.014
9	herd.1 targ.2 dist.	0.020	herd.1 targ.2 dist.	0.011	herd. targ.2 dist.	0.010	herd. targ.2 rel angle	0.012	herd.1 targ.2 dist.	0.014
10	targ.3 velocity	0.020	herd. targ.2 dist.	0.011	herd. accel.	0.010	herd. targ.3 rel angle	0.010	targ.3 velocity	0.013
	$T_{hor} = 32$ (1280 ms) prediction horizon									
	Label 0		Label 1		Label 2		Label 3		Label 4	
	Features	SHAP values	Features	SHAP values	Features	SHAP values	Features	SHAP values	Features	SHAP values
	Features	SHAP values	Features	SHAP values	Features	SHAP values	Features	SHAP values	Features	SHAP values
1	herd. targ.3 dist.	0.029	herd. targ.0 dist.	0.026	herd. targ.1 dist.	0.026	herd. targ.2 dist.	0.022	herd.1 targ.3 dist.	0.025
2	targ.1 velocity	0.028	herd.1 targ.0 dist.	0.021	herd.1 targ.1 dist.	0.016	herd. targ.3 dist.	0.018	herd. targ.3 dist.	0.019
3	targ.3 velocity	0.027	targ.0 direction	0.020	targ.1 goal dist.	0.016	herd.1 targ.2 dist.	0.018	herd.targ.0 dist.	0.016
4	herd.targ.0 dist.	0.026	herd.targ.3 dist.	0.016	herd.targ.3 dist.	0.016	targ.2 direction	0.016	targ.3 goal dist.	0.016
5	h goal dist.	0.025	herd. targ.1 dist.	0.015	herd. targ.2 dist.	0.015	herd.1 targ.3 dist.	0.016	herd. targ.2 dist.	0.013
6	herd.1 targ.3 dist.	0.024	herd.1 targ.3 dist.	0.014	herd. targ.0 dist.	0.014	herd. targ.0 dist.	0.016	targ.3 direction	0.012
7	herd. targ.2 dist.	0.024	herd. targ.2 dist.	0.014	targ.1 velocity	0.013	herd. targ.1 dist.	0.015	herd. targ.1 dist.	0.011
8	targ.2 velocity	0.023	targ.0 goal dist.	0.012	herd.1 targ.3 dist.	0.012	targ.2 goal dist.	0.015	herd.1 targ.0 dist.	0.011
9	herd. targ.1 dist.	0.022	herd. targ.3 rel angle	0.011	targ.3 velocity	0.012	targ.1 goal targ.h	0.013	targ.3 velocity	0.011
10	targ.0 velocity	0.020	targ.1 velocity	0.011	targ.1 goal targ.h	0.012	targ.0 goal targ.h	0.013	herd. targ.3 rel angle	0.010

Table 6 T

op 10 ranked features and corresponding SHAP values for each class predicted by the model trained on *expert* pairs with a sequence $T_{seq} = 1$s and model trained on balanced data.

	$T_{hor} = 16$ (640 ms) prediction horizon									
	Label 0		Label 1		Label 2		Label 3		Label 4	
	Features	SHAP values	Features	SHAP values	Features	SHAP values	Features	SHAP values	Features	SHAP values
1	herd.1 targ.1 dist.	0.034	herd.1 targ.0 dist.	0.032	herd.1 targ.1 dist.	0.034	herd.1 targ.2 dist.	0.022	herd.1 targ.3 dist.	0.029
2	herd.1 targ.3 dist.	0.032	herd. targ.0 dist.	0.018	herd. targ.0 dist.	0.018	herd. targ.0 dist.	0.015	herd. targ.0 dist.	0.015
3	herd. targ.0 dist.	0.031	herd.1 targ.1 dist.	0.015	targ.1 goal dist.	0.014	herd. targ.2 dist.	0.012	herd. targ.3 dist.	0.014
4	herd.1 targ.2 dist.	0.028	herd. targ.1 dist.	0.014	herd. targ.1 dist.	0.013	herd.1 targ.1 dist.	0.011	herd. targ.2 dist.	0.013
5	herd. targ.2 dist.	0.027	herd. targ.3 dist.	0.013	herd. targ.2 dist.	0.012	herd.1 targ.0 dist.	0.011	targ.3 goal dist.	0.013
6	herd.1 targ.0 dist.	0.026	targ.0 goal dist.	0.012	herd.1 targ.0 dist.	0.012	herd. targ.3 dist.	0.011	targ.3 direction	0.012
7	targ.2 velocity	0.026	targ.0 direction	0.012	herd.1 targ.3 dist.	0.011	herd. targ.1 dist.	0.009	herd. targ.1 dist.	0.011
8	herd. targ.1 dist.	0.021	herd. targ.2 rel angle	0.012	targ.2 velocity	0.010	targ.2 velocity	0.009	herd.1 targ.1 dist.	0.011
9	targ.1 velocity	0.020	herd. targ.2 dist.	0.011	herd. targ.3 dist.	0.010	targ.0 direction	0.009	herd.1 targ.0 dist.	0.010
10	herd. targ.2 rel angle	0.019	herd.1 targ.3 dist.	0.011	herd.1 targ.2 dist.	0.009	herd.1 targ.3 dist.	0.008	herd.1 targ.2 dist.	0.010
	$T_{hor} = 32$ (1280 ms) prediction horizon									
	Label 0		Label 1		Label 2		Label 3		Label 4	
	Features	SHAP values	Features	SHAP values	Features	SHAP values	Features	SHAP values	Features	SHAP values
	Features	SHAP values	Features	SHAP values	Features	SHAP values	Features	SHAP values	Features	SHAP values
1	herd.1 targ.1 dist.	0.038	herd.1 targ.0 dist.	0.033	herd.1 targ.1 dist.	0.029	herd.1 targ.2 dist.	0.023	herd.1 targ.3 dist.	0.030
2	herd.1 targ.3 dist.	0.033	herd.1 targ.1 dist.	0.017	targ.1 goal dist.	0.016	herd. targ.0 dist.	0.016	herd.1 targ.1 dist.	0.016
3	herd.1 targ.0 dist.	0.031	herd. targ.2 dist.	0.015	herd.1 targ.0 dist.	0.015	herd.1 targ.1 dist.	0.013	targ.3 goal dist.	0.015
4	herd. targ.0 dist.	0.027	herd.1 targ.3 dist.	0.014	herd. targ.1 dist.	0.013	herd.1 targ.0 dist.	0.012	herd. targ.2 dist.	0.015
5	herd. targ.2 dist.	0.026	herd. targ.0 dist.	0.013	herd. targ.2 dist.	0.013	herd. targ.1 dist.	0.012	targ.3 direction	0.014
6	h goal dist.	0.023	herd. targ.3 dist.	0.012	herd. targ.3 dist.	0.012	herd. targ.3 dist.	0.011	herd.1 targ.0 dist.	0.014
7	herd.1 targ.2 dist.	0.022	targ.0 goal targ.h	0.012	herd.1 targ.2 dist.	0.012	herd.1 targ.3 dist.	0.010	herd. targ.3 dist.	0.014
8	targ.1 goal dist.	0.022	herd. targ.1 dist.	0.011	herd. targ.0 dist.	0.011	herd. targ.2 rel angle	0.010	herd. targ.1 dist.	0.013
9	herd. targ.3 dist.	0.021	herd.1 targ.2 dist.	0.011	herd.1 targ.3 dist.	0.011	targ.1 goal dist.	0.009	herd. targ.0 dist.	0.013
10	herd. targ.1 dist.	0.020	herd.1 targ.3 rel angle	0.010	h goal dist.	0.011	herd. targ.2 dist.	0.009	herd.1 targ.2 dist.	0.012

References

[1] Nalepka, P., Lamb, M., Kallen, R.W., Shockley, K., Chemero, A., Saltzman, E., Richardson, M.J.: Human social motor solutions for human–machine interaction in dynamical task contexts. Proceedings of the National Academy of Sciences **116**(4), 1437–1446 (2019)

[2] Rigoli, L.M., Nalepka, P., Douglas, H., Kallen, R.W., Hosking, S., Best, C., Saltzman, E., Richardson, M.J.: Employing models of human social motor behavior for artificial agent trainers. In: Proceedings of the 19th International Conference on Autonomous Agents and MultiAgent Systems, pp. 1134–1142 (2020)
